# Identification of *Veillonella* Species in the Tongue Biofilm by Using a Novel One-Step Polymerase Chain Reaction Method

**DOI:** 10.1371/journal.pone.0157516

**Published:** 2016-06-21

**Authors:** Izumi Mashima, Citra Fragrantia Theodorea, Boonyanit Thaweboon, Sroisiri Thaweboon, Futoshi Nakazawa

**Affiliations:** 1 Postdoctoral Fellow of Japan Society for the Promotion of Science, 5-3-1, Kouji-machi, Chiyoda-Ku, Tokyo, 102-0083, Japan; 2 Department of Oral Microbiology, School of Dentistry, Health Sciences University of Hokkaido, 1757 Kanazawa, Ishikari-Tobetsu, Hokkaido, 061-0293, Japan; 3 Department of Oral Biology, Faculty of Dentistry, University of Indonesia, Jalan Salemba Raya No. 4, Jakarta, 10430, Indonesia; 4 Department of Oral Microbiology, Faculty of Dentistry, Mahidol University, 6 Yothi Street, Bangkok, 10400, Thailand; University of Malaya, MALAYSIA

## Abstract

Six *Veillonella* species have been frequently isolated from human oral cavities including infectious sites. Recently, it was reported that diet, smoking, and possibly socioeconomic status can influence the bacterial profile in oral cavities. In addition, oral hygiene habits may also influence oral microbiota in terms of both numbers and diversity of microorganisms. In this study, the identification of *Veillonella* species in tongue biofilms of Thai children, divided into three groups dependent on their status of oral hygiene. For this, we used a novel one-step PCR method with species-specific primer sets based on sequences of the *rpoB* gene. As shown in the results, the number of isolates of *Veillonella* species was 101 strains from only 10 of 89 subjects. However, the total number of bacteria was high for all subjects. Since it was reported in previous studies that *Veillonella* species were easy to isolate in human tongue biofilms at high numbers, the results obtained in this study may suggest country- or age-specific differences. Moreover, *Veillonella* species were detected predominantly in subjects who had poor oral hygiene compared to those with good or moderate oral hygiene. From these results, there is a possibility that *Veillonella* species may be an index of oral hygiene status. Furthermore, *V*. *rogosae* was a predominant species in tongue biofilms of Thai children, whereas *V*. *parvula* and *V*. *denticariosi* were not isolated at all. These characteristics of the distribution and frequency of *Veillonella* species are similar to those reported in previous studies. Although further studies are needed in other countries, in this study, a successful novel one-step PCR method was established to detect *Veillonella* species in human oral cavities easily and effectively. Furthermore, this is the first report investigating the distribution and frequency of *Veillonella* species in tongue biofilms of Thai children.

## Introduction

The genus *Veillonella* consists of small, strictly anaerobic, Gram-negative cocci that lack flagella, spores, and capsules. Members of this genus obtain energy from the utilization of short chain organic acids and have been isolated from the oral cavity and intestinal tract of humans and other animals [1, 2]. Currently, the genus *Veillonella* is subdivided into 13 species. Of these species, only *V*. *atypica*, *V*. *denticariosi*, *V*. *dispar*, *V*. *parvula*, *V*. *rogosae*, and *V*. *tobetsuensis* have been isolated from human oral cavities [3–7]. The main habitats of these oral *Veillonella* are the tongue, buccal mucosa, and saliva [3, 8–12]. Oral *Veillonella*, especially *V*. *parvula*, are associated with severe early childhood caries [13] and intraradicular infections [14, 15], including abscesses [16], apical root canals [17], and dental tubules [18]. *Veillonella* species are also predominantly found in subgingival biofilm samples of patients who have chronic periodontitis [19–21], and our previous study concluded that *V*. *parvula* is associated with the state of chronic periodontitis [21]. Furthermore, in periodontal patients undergoing therapy, *Veillonella* species, along with *Streptococcus* and *Neisseria* species, were found to be consistently resistant to tetracycline [22]. Moreover, tetracycline-resistant *Veillonella* species have the opportunity to come in close contact with, and consequently transfer resistance elements to other oral bacteria and bacteria that pass through the oral cavity [23]. *Veillonella* species were previously known to be sensitive to penicillin and ampicillin but are now frequently resistant to these antibiotics [24]. In addition, Delwiche et al. [25] reported that *Veillonella* species produce a large amount of lipopolysaccharides (LPS) and Metera et al. [26] reported that with *V*. *parvula*, LPS-stimulated cytokine induction, as well as p38 MAPK activation, are Toll-like receptor 4-dependent. It is thought that these properties of *Veillonella* species make it difficult to treat the associated periodontitis.

In the case of dental caries, *Veillonella* species are highly associated with lactic acid-producing species [27]. This is not surprising given their reliance on lactate as a nutrient source. This has potential clinical utility; *Veillonella* levels may serve as a sensitive biological indicator and early warning sign of acid production. In addition, among children without a history of caries, the presence of *Veillonella* or other acid-producing bacteria, including *Streptococcus mutans*, has predicted the development of future caries [27].

The bacterial communities found in the human oral cavities, with around 1000 species present [28], have been shown to be the second most complex in the body, after the colon [29]. It has been known for a long time that periodontitis and dental caries are caused by oral biofilms. Oral biofilms are composed of multiple species, whose development is initiated by adherence of pioneer species to the salivary proteins and glycoproteins adsorbed onto the tooth enamel. The biofilm is not formed by the random simultaneous colonization of these species, but rather by selective, reproducible, and sequential colonization [30, 31].

As mentioned above, it is evident that oral *Veillonella* are associated with oral biofilms, which cause many human oral infectious diseases, such as periodontitis and dental caries. Furthermore, Periasamy and Kolenbrander [32] reported that *Veillonella* species had a central role as an early colonizer in establishing multispecies oral biofilm communities having initial, middle, and late colonizers. Therefore, it would appear that understanding the distribution and frequency of *Veillonella* species in oral biofilms is important in considering treatments or preventions for these oral infectious diseases. Furthermore, several recent reports indicated that diet, smoking, and potentially socioeconomic status can influence the bacterial profile of oral cavities [33–35]. According to these reports, the possibility of having country-, community-, or family-specific distribution and frequency of bacteria in oral biofilms has been considered. In addition, it was reported that oral hygiene habits might also influence oral microbiota qualitatively and quantitatively [36, 37].

In this study, the distribution and frequency of *Veillonella* species in tongue biofilms of children in Thailand, in association with the level of oral hygiene, were examined. We then compared the results to previous reports of our group and others regarding the identification of *Veillonella* species in tongue biofilm. Moreover, the effective novel one-step PCR method using species-specific primer sets designed from the *rpo B* gene (encoding the β subunit of bacterial RNA polymerase) was established in this study.

## Materials and Methods

### Ethics Statement

This study received approval from the Ethics Committee, Mahidol University, Bangkok, Thailand under process number MU-DT/PY-IRB 2015/DT028, and samples were collected in March 2015. The participants and their parents were made aware of the objectives and procedures of the study and agreed to participate by providing written, informed consent.

### Subjects

The 89 children selected consisted of 41 males and 48 females (age range: 7 to 15 years). Children with a history of immunosuppression or systemic diseases (e.g. diabetes and HIV), those using medications that reduce saliva flow, and those under treatment with antimicrobials in the previous three months, were excluded from the study. The participants were evaluated by the Simplified Oral Hygiene Index (OHI-S) according to the criteria of Greene &Vermillion [38] and divided into three groups. The first group (Good oral hygiene) was composed of 29 children (10 males and 19 females) with OHI-S scores of 0–1.2. The second group (Moderate oral hygiene) was composed of 30 children (15 males and 15 females) with OHI-S scores of 1.3–3.0. The third group (Poor oral hygiene) was composed of 30 children (16 males and 14 females) with OHI-S scores of 3.1–6.0.

### Sample Collection

Tongue biofilm samples were collected at Dental Hospital, Mahidol University Faculty of Dentistry using sterile cotton wool swabs to swab the tongue 5 times each per subject. Samples in reduced transport fluids were transported in an anaerobic box (HIRASAWA WORKS Inc.) containing 80% N_2_, 10% CO_2_, and 10% H_2_. The samples were immediately placed in 1 mL of sterile saline (< 1 hour from the time of collection). Samples were homogenized for 3 min with a BioMasher^®^II(Nippi, Incorporated Protein Engineering Office, Tokyo, Japan) to disperse the biofilm and were serially diluted 10-fold with sterile saline from 10^−3^ to 10^−8^.

### Culture Conditions

Aliquots of serial 10-fold dilutions (100 μL) were used to inoculate Bacto^™^ Brain Heart Infusion (Difco Laboratories, BD) supplemented with 5% (volume/volume) defibrinated sheep blood (BHI agar), hemin (10 μg/mL), and menadione (5 μg/mL), and also the selective medium, *Veillonella* agar [39]. After inoculation, all media were incubated in an anaerobic box containing 80% N_2_, 10% CO_2_, and 10% H_2_ at 37°C; *Veillonella* agar was incubated for 5 days, while BHI agar was incubated for 7 days.

The total number of bacteria in the samples was determined by counting the total number of colonies on BHI agar, while the number of *Veillonella* was determined by counting the total number of typical *Veillonella* colonies on *Veillonella* agar. Bacterial cells of typical *Veillonella* colonies were confirmed by light microscopy after Gram staining.

### Bacterial Strains

*V*. *atypica* ATCC 17744^T^, *V*. *denticariosi* JCM 15641^T^, *V*. *dispar* ATCC 17748^T^, *V*. *parvula* ATCC 10790^T^, *V*. *rogosae* JCM 15642^T^ and *V*. *tobetsuensis* ATCC BAA-2400 (= JCM 17976^T^) were used as control oral *Veillonella* to confirm the specificity of the primer sets. They were cultured on BHI agar at 37°C in the anaerobic box for 5 days.

### DNA Extraction

Genomic DNA was extracted from individual bacterial cells using an InstaGene Matrix Kit (Bio-Rad) and the DNA concentration was determined based on fluorescence using a Qubit^®^ 3.0 Fluorometer (Invitrogen life technologies) according to the manufacturer’s instructions.

### Design of Primer Sets

According to the DNA sequence of a conserved region of the partial sequence of the *rpo B* gene, species-specific primer sets for six *Veillonella* species were designed by the standard manual method [40]. The accession numbers of the *rpo B* gene sequences of reference strains from the GenBank database (Database ID: BA123456) were EF185159 for *V*. *atypica*, EF185162 for *V*. *denticariosi*, EF185161 for *V*. *dispar*, EF185158 for *V*. *parvula*, EF211831 for *V*. *rogosae*, and AB698646 for *V*. *tobetsuensis*. Pairwise similarities among six *Veillonella* species were estimated by MEGALINE including CLUSTAL W in the LASERGENE program (DNASTAR).

### Identification of *Veillonella* Species

Specific primer sets were used to obtain the PCR products described below. For identification of oral *Veillonella* at the genus level, one primer pair: Veill-rpoBF (5’-GTAACAAAGGTGTCGTTTCTCG-3’) and Veill-rpoBR (5’-GCACCRTCAAATACAGGTGTAGC-3’) was used before identification of oral *Veillonella* at the species level [41, 42].

### PCR Protocol

PCR at the species level was performed using 1 μL of template DNA, 1 μL of each primer (10 pmol/mL), 17 μL of PCR grade water, and 25 μL of master mix from an AmpliTaq Gold^®^ 360 Master Mix (Applied Biosystems). When using specific primer sets for six *Veillonella* species, PCR mixtures were subjected to preheating at 94°C for 15 min; followed by 30 cycles of 92°C for 30 s, annealing at 55°C for 30 s, and extension at 72°C for 30 s; with a final extension at 72°C for 5 min. The PCR products were applied to a 3.0% agarose gel. In the case of PCR at the genus level, the PCR protocol was performed in accordance with the protocols described by Arif et al. and Beighton et al. [6, 41]. After electrophoresis, the gel was stained with SYBR^®^ Safe DNA gel stain (Invitrogen^™^).

### Clonal Analysis of Unknown Strains

Unknown strains of genus *Veillonella* (PCR reaction was positive with genus-specific primers, but was negative with species-specific primer sets in this study) were examined clonal analysis. The representative 11 strains in the 40 unknown strains were chosen in this study.

DNA was extracted from the individual bacterial cells isolated on *Veillonella* agar by using the InstaGene Matrix kit (Bio-Rad), according to the manufacturer’s instructions. PCR-based amplification and partial sequence analyses of *rpoB* were performed using previously described specific primers for genus *Veilloenlla* [41, 42]. The sequence determined with an ABI PRISM 310 Genetic Analyzer were aligned with each other and connected by using SEQMAN IIof the LASERGENE program (DNASTAR). The programs MEGALIGN including CLUSTALW and NJPlot were used to compare sequences and the reconstruct the evolutionary tree by the neighbour-joining method. Also, confidence intervals were assessed by CLUSTAL W with bootstrap analysis. In particular, pairwise similarity values were determined with MEGALIGN in the LASERGENE program. The *rpoB* (559 nt) partial sequences of 11 strains were aligned against the sequences of the representative strains retrieved from GenBank.

## Results

### Species-specific Primer Sets for Oral *Veillonella*

A similarity search of the *rpo B* gene of six *Veillonella* species revealed a high degree of polymorphism in the region of position 2500 to 3100 in all oral *Veillonella* tested. The similarities among partial sequences of the *rpo B* gene regions of these six *Veillonella* species were found to be from 73.7 to 90.9% (Table 1).

**Table 1:** Level of *rpoB* partial sequence similarity among six species of oral *Veillonella*.

	Percentage of similarity with					
Species	*V*. *atypica*	*V*. *denticariosi*	*V*. *dispar*	*V*. *parvula*	*V*. *rogosae*	*V*. *tobetsuensis*
***V*. *atypica***						
***V*. *denticariosi***	73.7					
***V*. *dispar***	81.4	76.6				
***V*. *parvula***	76.7	78.2	82.8			
***V*. *rogosae***	75.8	81	83.2	90.9		
***V*. *tobetsuensis***	80.7	74.1	81.9	76.9	78.5	

**Fig 1:**
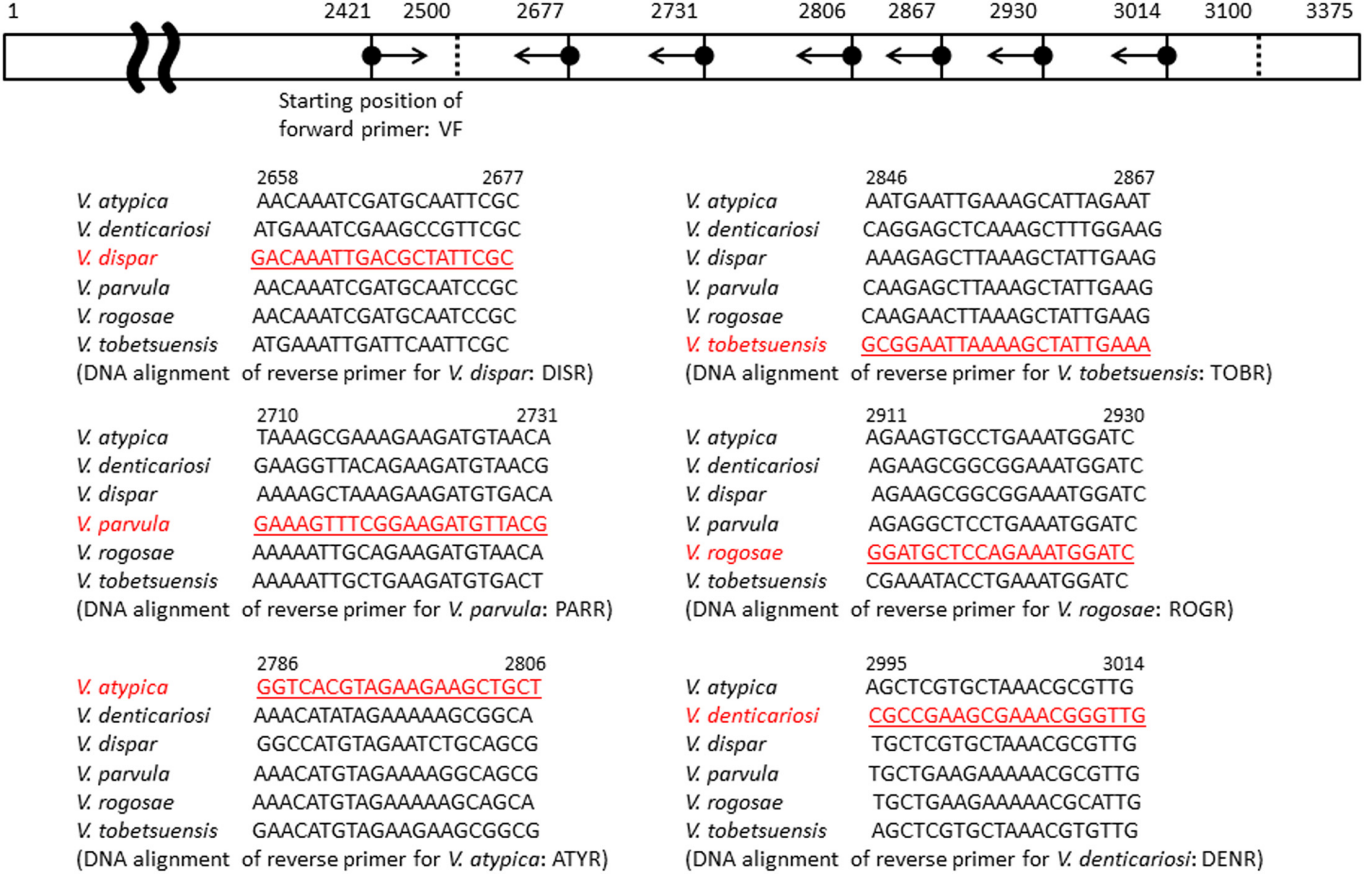
The location and sequence of species-specific primers for the *rpoB* gene of oral *Veillonella*. Underlined red font indicates the nucleotide sequences of each reverse primer for different *Veillonella* species. Numbers indicate the nucleotide positions in the *rpoB* gene of *Veillonella* species. Black font indicates sequences of the *rpoB* gene of other species of *Veillonella* corresponding to the same nucleotide positions of the reverse primers indicated.

One forward primer, VF (5’-GTAACAAAGGTGTCGTTTCTCG-3’), was designed using the sequence of the conserved region of the *rpo B* gene for all *Veillonella* species (Fig 1). According to the sequences of the variable regions in the *rpo B* gene of oral *Veillonella*, six reverse primers were designed as species-specific primers. These specific reverse primers were designated as ATYR (5’-AGCAGCTTCTTCTACGTGACC-3’) for *V*. *atypica*, as DENR (5’-CAACCCGTTTCGCTTCGGCG-3’) for *V*. *denticariosi*, as DISR (5’-GCGAATAGCGTCAATTTGTC-3’) for *V*. *dispar*, as PARR (5’-CGTAACATCTTCCGAAACTTTC-3’) for *V*. *parvula*, as ROGR (5’-GATCCATTTCTGGAGCATCC-3’) for *V*. *rogosae* and as TOBR (5’-TTTCAATAGCTTTTAATTCCGC-3’) for *V*. *tobetsuensis* (Fig 1).

### Specificity and Sensitivity of the Primer Sets

**Fig 2:**
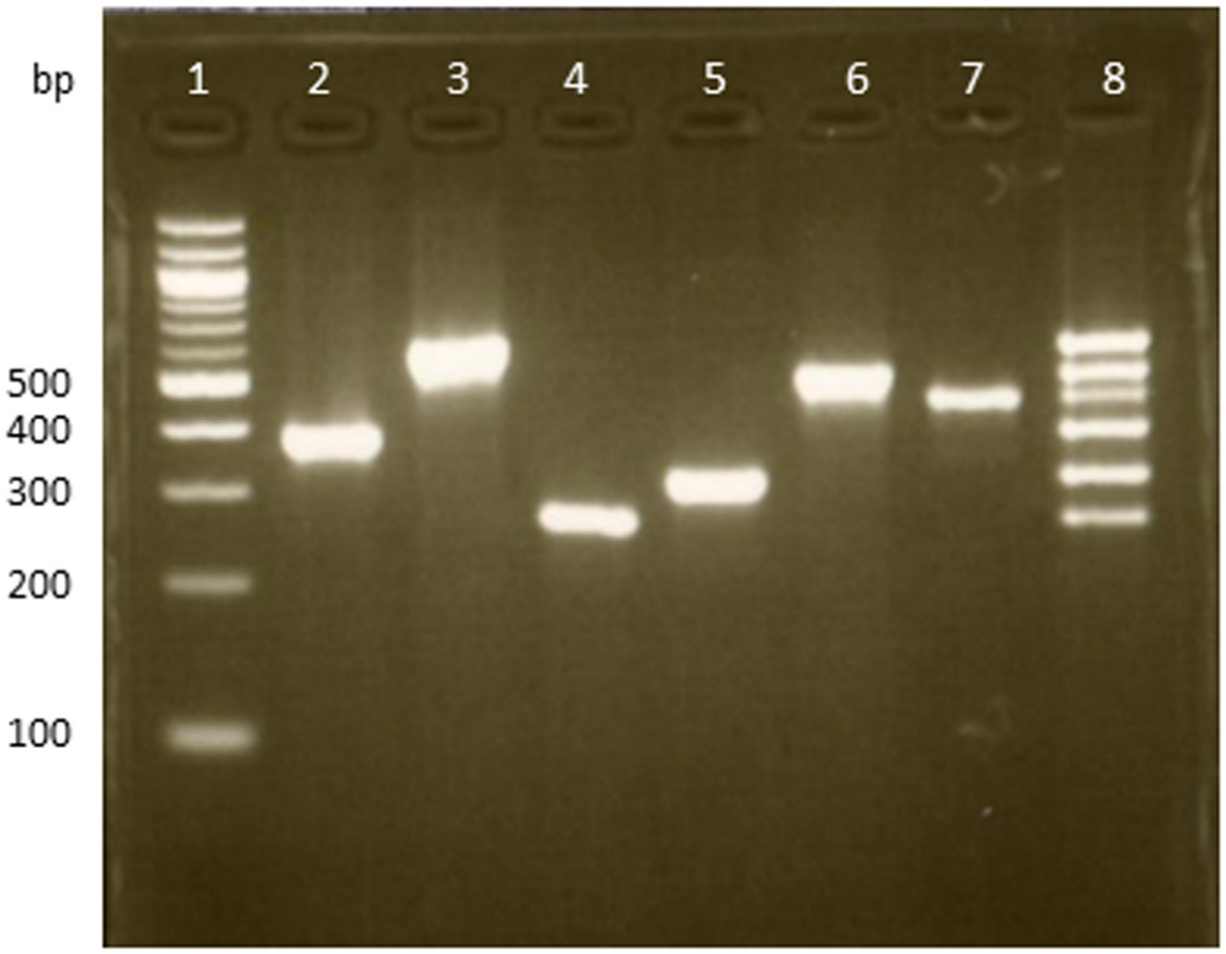
The products of the polymerase chain reaction obtained by using a mixture of primers. Molecular weight marker is in lane 1. Primers were a mixture of VF, ATYR, DENR, DISR, PARR, ROGR, and TOBR. Template DNA was from *V*. *atypica*, lane 2; *V*. *denticariosi*, lane 3; *V*. *dispar*, lane 4; *V*. *parvula*, lane 5; *V*. *rogosae*, lane 6; *V*. *tobetsuensis*, lane 7; the mixture of all PCR products of *Veillonella* species, lane 8.

To achieve faster and more convenient identification of oral *Veillonella*, all six reverse primers (ATYR, DENR, DISR, PARR, ROGR, and TOBR) were examined for use with VF in single PCR mixture. PCR was performed using DNA templates from the type strain of six *Veillonella* species as described in Materials and Methods. The electrophoretically detected PCR amplicons were shown as species-specific products (Fig 2). The molecular weights of the PCR products were also identical to the theoretical values, which were 396 base pairs (bp) for *V*. *atypica*, 594 bp for *V*. *denticariosi*, 257 bp for *V*. *dispar*, 311 bp for *V*. *parvula*, 510 bp for *V*. *rogosae* and 447 bp for *V*. *tobetsuensis* (Fig 2).

Each of the six PCR products were mixed and applied to the agarose gel to use as a molecular marker (Fig 2) for reference when six *Veillonella* species were identified from tongue samples, through electrophoresis. These results demonstrated that six *Veillonella* species could be specifically and effectively identified by PCR using a mixture of primer sets, ATYR, DENR, DISR, PARR, ROGR, TOBR and VF.

**Fig 3:**
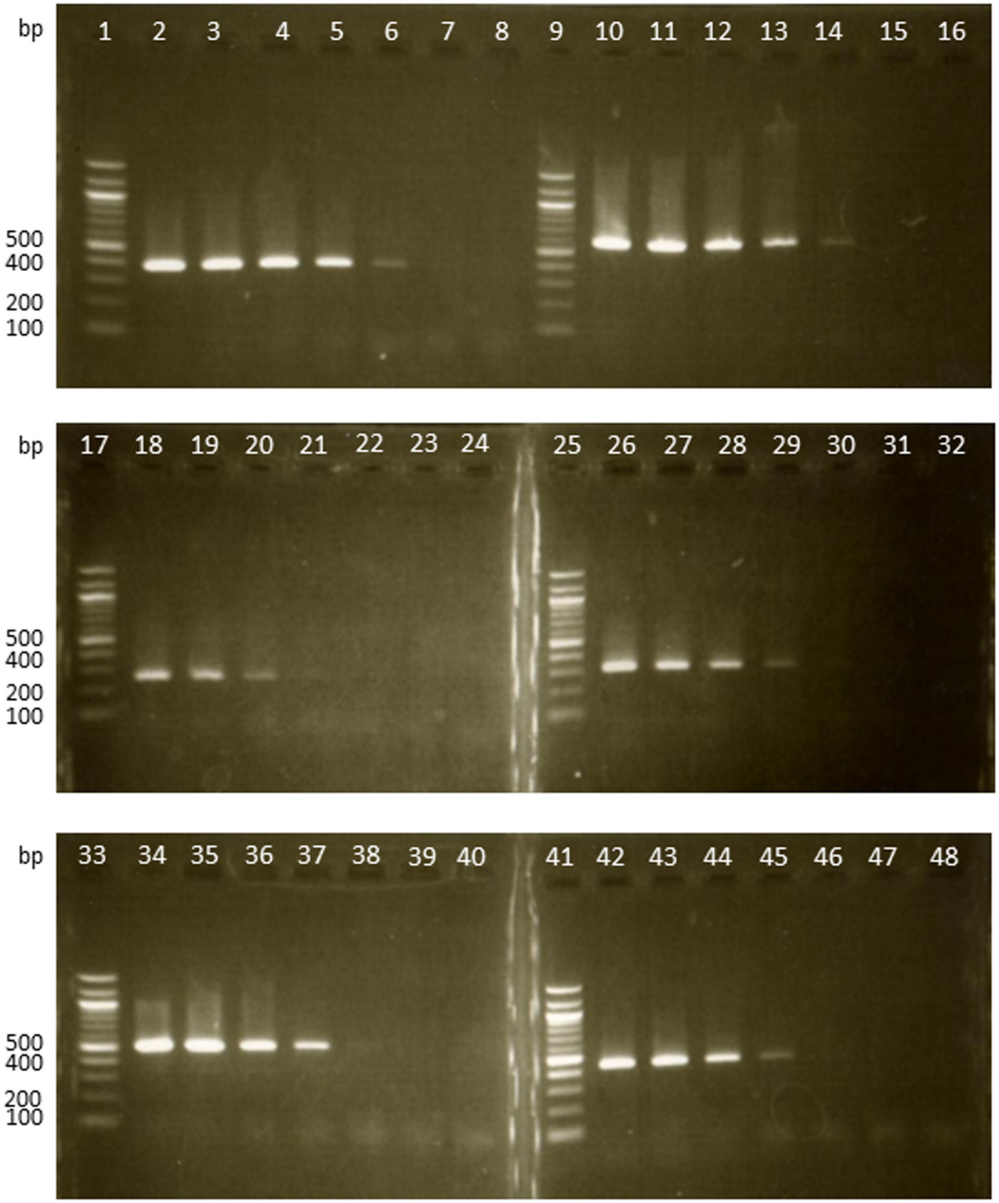
Sensitivity of the PCR for decreasing concentrations of genomic DNA purified from *Veillonella* species using species-specific primer sets. A molecular weight marker is shown in lanes 1, 9, 17, 25, 33, and 41. Lane 2 to lane 8: *V*. *atypica*, lane 10 to 16: *V*. *denticariosi*, lane 18 to 24: *V*. *dispar*, lane 26 to 32: *V*. *parvula*, lane 34 to 40: *V*. *rogosae*, lane 42 to 48: *V*. *tobetsuensis*. Amount of the target genomic DNA in the reaction mixture is as follows. Lane 2, 10, 18, 26, 34, and 42: 10 ng; lane 3, 11, 19, 27, 35, and 43: 1 ng; lane 4, 12, 20, 28, 36, and 44: 0.1 ng, lane 5, 13, 21, 29, 38, and 45: 0.01 ng; lane 6, 14, 22, 30, 38, and 46: 1 pg; lane 7, 15, 23, 31, 39, and 47: 0.1 pg; lane 8, 16, 24, 32, 40, and 48: 0.01 pg.

To examine the sensitivity of these primers, PCR was performed with 0.01pg– 10 ng of template DNA from the type strains of six *Veillonella* species (Fig 3). 1 pg of *V*. *atypica* and *V*. *denticariosi* DNA, 0.1–0.01 ng of *V*. *dispar* DNA, 0.01 ng of *V*. *parvula* and *V*. *tobetsuensis* DNA, and 0.01 ng- 1 pg of *V*. *rogosae* DNA could be detected using these primer sets (Fig 3).

### Detection of Oral *Veillonella* at the Species Level from Tongue Samples

Oral *Veillonella* were detected in only 10 subjects from 89 subjects from all oral hygiene groups as shown in Tables 2–4. However, the tongue biofilm swabs did indeed yield high numbers of bacterial colonies on BHI agar. Mean (SE) colony-forming units (CFU/mL) per swab were 2.2 (0.08) x 10^6^ with a median of 9.0 x 10^5^ in the first group (Good oral hygiene), Table 2, 5.6 (0.02) x 10^5^ with a median of 9.0 x 10^4^ in the second group (Moderate oral hygiene), Table 3, and 1.4 (0.04) x 10^6^ with a median of 6.5 x 10^5^ in the third group (Poor oral hygiene), Table 4. A total of 101 strains were isolated from *Veillonella* agar in all groups. *Veillonella* species were predominantly detected in subjects who had poor oral hygiene compared to those with good or moderate oral hygiene (Tables 2–4). Typical *Veillonella* colonies on the *Veillonella* agar were 2–4 mm in diameter, regular and slightly domed in shape and were entirely white; they were composed of small, gram-negative coccal cells, mainly existing as single cells but with some short chains visible. The detection limit was dependent on the number of bacteria in the sample and was < 0.1% of the total colony count.

**Table 2:** The first group (Good oral hygiene).

The first group (Good oral hygiene [OHIs 0–1.2])												
Subject	Age	Sex	Total number		Isolated *Veillonella* spp.							
			All bacteria CFU/mL (× 10^7^)	*Veillonella* spp. CFU/mL (× 10^3^)	Total number (100%)	*V*. *atypica* number (%)	*V*. *denticariosi* number (%)	*V*. *dispar* number (%)	*V*. *parvula* number (%)	*V*. *rogosae* number (%)	*V*. *tobetsuensis* number (%)	Unknown species number (%)
G-1	10	F	2.4	0.006	6	0 (0.0)	0 (0.0)	2 (33.3)	0 (0.0)	2 (33.3)	0 (0.0)	2 (33.3)
G-2	11	F	0.02	0.009	9	0 (0.0)	0 (0.0)	0 (0.0)	0 (0.0)	1 (11.1)	0 (0.0)	8 (88.9)
G-3	10	M	0.03	0	-	-	-	-	-	-	-	-
G-4	9	F	0.002	0	-	-	-	-	-	-	-	-
G-5	12	M	0.09	0	-	-	-	-	-	-	-	-
G-6	15	F	0.002	0	-	-	-	-	-	-	-	-
G-7	13	M	0.3	0	-	-	-	-	-	-	-	-
G-8	11	F	0.006	0	-	-	-	-	-	-	-	-
G-9	11	M	0.4	0	-	-	-	-	-	-	-	-
G-10	10	F	0.02	0	-	-	-	-	-	-	-	-
G-11	11	M	0.1	0	-	-	-	-	-	-	-	-
G-12	10	F	0.2	0	-	-	-	-	-	-	-	-
G-13	10	M	0.03	0	-	-	-	-	-	-	-	-
G-14	10	F	0.002	0	-	-	-	-	-	-	-	-
G-15	11	F	0.06	0	-	-	-	-	-	-	-	-
G-16	12	F	0.4	0	-	-	-	-	-	-	-	-
G-17	10	F	0.3	0	-	-	-	-	-	-	-	-
G-18	9	F	0.1	0	-	-	-	-	-	-	-	-
G-19	9	F	0.01	0	-	-	-	-	-	-	-	-
G-20	12	M	0.005	0	-	-	-	-	-	-	-	-
G-21	8	F	0.5	0	-	-	-	-	-	-	-	-
G-22	13	M	0.01	0	-	-	-	-	-	-	-	-
G-23	12	M	0.1	0	-	-	-	-	-	-	-	-
G-24	11	M	0.2	0	-	-	-	-	-	-	-	-
G-25	13	F	0.5	0	-	-	-	-	-	-	-	-
G-26	9	F	0.06	0	-	-	-	-	-	-	-	-
G-27	7	F	0.2	0	-	-	-	-	-	-	-	-
G-28	10	F	0.2	0	-	-	-	-	-	-	-	-
G-29	10	F	0.08	0	-	-	-	-	-	-	-	-

Total number of anaerobic bacterial colony counts, total number of *Veillonella* species counts and the number of isolates from each subject identified using species-specific primer sets. CFU: colony-forming unit; detection limit <0.1% of the total count. Individual species as a percentage of the number of isolates from each subject identified using species-specific primer sets.

**Table 3:** The second group (Moderate oral hygiene).

The second group (Moderate oral hygiene [OHIs 1.3–3.0])												
Subject	Age	Sex	Total number		Isolated *Veillonella* spp.							
			All bacteria CFU/mL (× 10^7^)	*Veillonella* spp. CFU/mL (× 10^3^)	Total number (100%)	*V*. *atypica* number (%)	*V*. *denticariosi* number (%)	*V*. *dispar* number (%)	*V*. *parvula* number (%)	*V*. *rogosae* number (%)	*V*. *tobetsuensis* number (%)	Unknown species number (%)
M-1	11	F	0.3	2.0	20	2 (10.0)	0 (0.0)	4 (20.0)	0 (0.0)	9 (45.0)	0 (0.0)	5 (25.0)
M-2	12	F	0.2	2.0	20	6 (30.0)	0 (0.0)	1 (5.0)	0 (0.0)	0 (0.0)	1 (5.0)	12 (60.0)
M-3	10	M	0.05	0	-	-	-	-	-	-	-	-
M-4	13	F	0.004	0	-	-	-	-	-	-	-	-
M-5	12	M	0.0004	0	-	-	-	-	-	-	-	-
M-6	11	M	0.001	0	-	-	-	-	-	-	-	-
M-7	10	M	0.08	0	-	-	-	-	-	-	-	-
M-8	9	F	0.001	0	-	-	-	-	-	-	-	-
M-9	10	M	0.04	0	-	-	-	-	-	-	-	-
M-10	11	F	0.06	0	-	-	-	-	-	-	-	-
M-11	10	M	0.4	0	-	-	-	-	-	-	-	-
M-12	11	F	0.08	0	-	-	-	-	-	-	-	-
M-13	10	M	0.06	0	-	-	-	-	-	-	-	-
M-14	12	M	0.03	0	-	-	-	-	-	-	-	-
M-15	12	M	0.004	0	-	-	-	-	-	-	-	-
M-16	11	F	0.03	0	-	-	-	-	-	-	-	-
M-17	13	M	0.001	0	-	-	-	-	-	-	-	-
M-18	11	F	0.008	0	-	-	-	-	-	-	-	-
M-19	11	F	0.0005	0	-	-	-	-	-	-	-	-
M-20	9	F	0.002	0	-	-	-	-	-	-	-	-
M-21	12	F	0.2	0	-	-	-	-	-	-	-	-
M-22	11	M	0.0004	0	-	-	-	-	-	-	-	-
M-23	11	F	0.01	0	-	-	-	-	-	-	-	-
M-24	11	F	0.0001	0	-	-	-	-	-	-	-	-
M-25	11	M	0.003	0	-	-	-	-	-	-	-	-
M-26	11	M	0.008	0	-	-	-	-	-	-	-	-
M-27	11	M	0.005	0	-	-	-	-	-	-	-	-
M-28	11	F	0.03	0	-	-	-	-	-	-	-	-
M-29	9	F	0.09	0	-	-	-	-	-	-	-	-
M-30	15	M	0.001	0	-	-	-	-	-	-	-	-

Total number of anaerobic bacterial colony counts, total number of *Veillonella* species counts and the number of isolates from each subject identified using species-specific primer sets. CFU: colony-forming unit; detection limit <0.1% of the total count. Individual species as a percentage of the number of isolates from each subject identified using species-specific primer sets.

**Table 4:** The third group (Poor oral hygiene).

The third group (Poor oral hygiene [OHIs 3.1–6.0])												
Subject	Age	Sex	Total number		Isolated *Veillonella* spp.							
			All bacteria CFU/mL (× 10^7^)	*Veillonella* spp. CFU/mL (× 10^3^)	Total number (100%)	*V*. *atypica* number (%)	*V*. *denticariosi* number (%)	*V*. *dispar* number (%)	*V*. *parvula* number (%)	*V*. *rogosae* number (%)	*V*. *tobetsuensis* number (%)	Unknown species number (%)
P-1	11	F	0.07	0.002	2	0 (0.0)	0 (0.0)	0 (0.0)	0 (0.0)	1 (50.0)	0 (0.0)	1 (50.0)
P-2	9	M	0.02	2.0	20	0 (0.0)	0 (0.0)	0 (0.0)	0 (0.0)	10 (50.0)	0 (0.0)	10 (50.0)
P-3	13	M	0.1	0.002	2	1 (50.0)	0 (0.0)	0 (0.0)	0 (0.0)	1 (50.0)	0 (0.0)	0 (0.0)
P-4	12	M	0.7	0.001	1	0 (0.0)	0 (0.0)	0 (0.0)	0 (0.0)	1 (100.0)	0 (0.0)	0 (0.0)
P-5	11	M	0.1	2.0	20	0 (0.0)	0 (0.0)	0 (0.0)	0 (0.0)	19 (95.0)	0 (0.0)	1 (5.0)
P-6	9	M	0.1	0.001	1	0 (0.0)	0 (0.0)	0 (0.0)	0 (0.0)	0 (0.0)	0 (0.0)	1 (100.0)
P-7	9	M	0.06	0	-	-	-	-	-	-	-	-
P-8	12	F	0.4	0	-	-	-	-	-	-	-	-
P-9	10	M	0.03	0	-	-	-	-	-	-	-	-
P-10	11	F	0.1	0	-	-	-	-	-	-	-	-
P-11	11	F	0.01	0	-	-	-	-	-	-	-	-
P-12	11	F	0.005	0	-	-	-	-	-	-	-	-
P-13	11	M	0.05	0	-	-	-	-	-	-	-	-
P-14	10	F	0.01	0	-	-	-	-	-	-	-	-
P-15	13	F	0.01	0	-	-	-	-	-	-	-	-
P-16	11	F	0.2	0	-	-	-	-	-	-	-	-
P-17	10	F	0.5	0	-	-	-	-	-	-	-	-
P-18	11	F	0.06	0	-	-	-	-	-	-	-	-
P-19	11	M	0.2	0	-	-	-	-	-	-	-	-
P-20	9	F	0.2	0	-	-	-	-	-	-	-	-
P-21	11	M	0.08	0	-	-	-	-	-	-	-	-
P-22	9	F	1.0	0	-	-	-	-	-	-	-	-
P-23	9	M	0.004	0	-	-	-	-	-	-	-	-
P-24	12	M	0.005	0	-	-	-	-	-	-	-	-
P-25	11	F	0.08	0	-	-	-	-	-	-	-	-
P-26	7	M	0.03	0	-	-	-	-	-	-	-	-
P-27	12	M	0.03	0	-	-	-	-	-	-	-	-
P-28	9	M	0.02	0	-	-	-	-	-	-	-	-
P-29	11	M	0.09	0	-	-	-	-	-	-	-	-
P-30	11	F	0.01	0	-	-	-	-	-	-	-	-

Total number of anaerobic bacterial colony counts, total number of *Veillonella* species counts and the number of isolates from each subject identified using species-specific primer sets. CFU: colony-forming unit; detection limit <0.1% of the total count. Individual species as a percentage of the number of isolates from each subject identified using species-specific primer sets.

Using species-specific primer sets, 61 of the 101 isolates were identified as either *V*. *atypica*, *V*. *dispar*, *V*. *rogosae*, or *V*. *tobetsuensis* (Tables 2–4). Among them, *V*. *rogosae* was detected as the predominant species through all groups. However, *V*. *parvula* and *V*. *denticariosi* were not isolated from any subjects. *V*. *dispar* was isolated mainly from subjects who had good or moderate oral hygiene. In addition, of the 101 strains isolated in this study, zero or multiple PCR products were detected using species-specific primer sets with DNA from 40 strains isolated from eight subjects (Tables 2–4). These 40 strains made distinctive PCR products with *Veillonella* genus primers (Data not shown).

### Clonal Analysis of Unknown Strains

**Fig 4:**
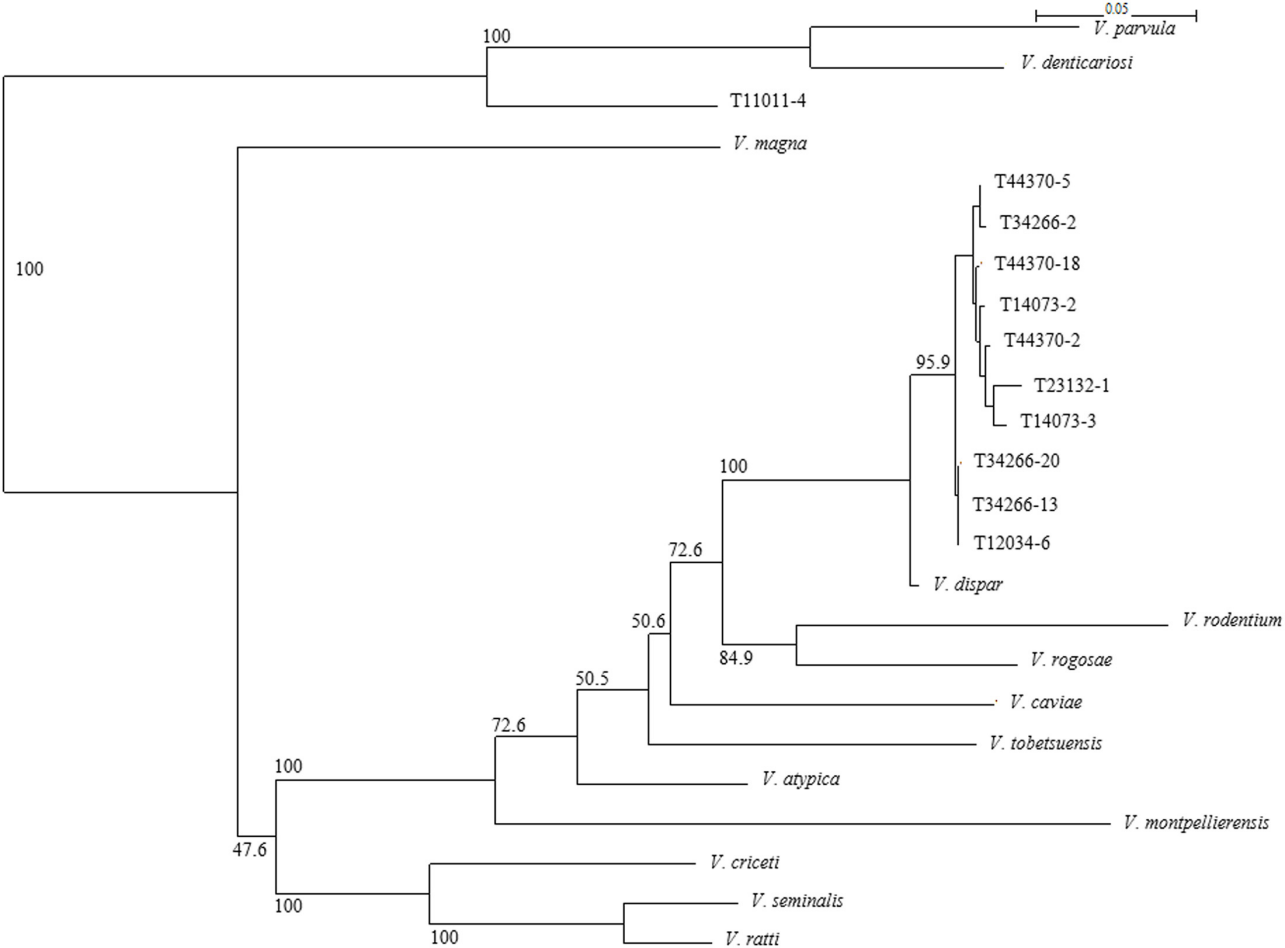
Neighbour-joining tree based on *rpoB* gene sequences (559nt), showing the relationship between the representative 11 unknown strains T11011-4, T44370-5, T34266-2, T44370-18, T14073-2, T44370-2, T23132-1, T14073-3, T34266-20, T34266-13 and T12034-6 and the strains of the recognized members of the genus *Veillonella*. The strain of each species and GenBank/EMBL/DDBJ accession numbers for *rpoB* gene sequences are shown as follows; *V*. *atypica* ATCC 17744^T^ (EF185159), *V*. *caviae* ATCC 33540^T^ (EF185163), *V*. *criceti* ATCC 17747^T^, *V*. *denticariosi* ATCC JCM 15641^T^ (EF185162), *V*. *dispar* ATCC 17748^T^ (EF185161), *V*. *magna* lac18^T^ (GU479075), *V*. *montpellierensis* ADV 281.99^T^ (EF411194), *V*. *parvula* ATCC 10790^T^ (EF185158), *V*. *ratti* ATCC 17746^T^ (EF185165), *V*. *rodentium* ATCC 17743^T^ (EF185166), *V*. *rogosae* JCM 15642^T^ (EF211831), *V*. *seminalis* strain 08/01/13-B-2228 (KJ580479), *V*. *tobetsuensis* ATCC BAA-2400^T^ (AB698646), ^T^; Type strain. Bootstrap values are indicated at corresponding nodes. Bar, 0.05 substitutions per site.

The 11 strains formed distinct taxa with robust bootstrap support (100 and 95.9%) in the *rpoB* tree (Fig 4) within the genus *Veillonella*. The *rpoB* partial sequence similarity among the 11 strains was 92.4–100%. The mean *rpoB* sequence similarity among the 11 strains belonged to *V*. *dispar* and the most closely related species was 98.3%.

## Discussion

*Veillonella* species are relatively easily identified at the genus level, but species-level identification of the members of this genus is difficult because of a lack of conventional phenotypic and biochemical tests [5, 42–44]. Molecular methods based on 16S ribosomal DNA (rDNA) gene sequencing, including PCR-random fragment length polymorphism analysis, have been used to identify *Veillonella* strains at the species level [44, 45]. However, recent studies have shown that identification of the members of this genus using 16S rDNA gene sequencing is not reliable [6, 7, 44, 46]. To overcome this problem, sequence analysis of housekeeping genes, including *rpoB*, *dnaK*, and *gyrB*, have been used to define *Veillonella* species [5–7, 46]. This approach has enabled clear discrimination between species that are well defined based on DNA-DNA homology but poorly defined based on 16S rDNA. *V*. *denticariosi*, *V*. *rogosae*, and *V*. *tobetsuensis* have been proposed as novel species of oral *Veillonella* based on the results of the molecular analysis using *rpoB* or *dnaK* gene sequencing in conjunction with sequence analysis of the 16S r DNA gene [5–7]. Beighton et al. [11] successfully used the *rpoB* gene sequence, rather than 16S rDNA gene sequence, to discriminate between 253 *Veillonella* isolates from human tongues of healthy adults at the species level. However, it is time consuming to obtain sequence data of many strains, and laborious to identify them at the species level.

Igarashi et al. [47] reported the successful design of species-specific primer sets for five species of oral *Veillonella* (*V*. *atypica*, *V*.*denticariosi*, *V*. *dispar*, *V*. *parvula*, and *V*. *rogosae*) based on a highly variable region (positions 2500–3100) of the *rpoB* gene. They distinguished between five species of oral *Veillonella* at the species level by using PCR products generated by a two-step PCR method with species-specific primer sets. This proved to be easier than using complete *rpoB* gene sequences. In addition, the distribution and frequency of five species of oral *Veillonella* in tongue biofilms of healthy adults were analyzed by PCR with these specific-primer sets as performed in our previous study [12]. Furthermore, species-specific primer pairs for *V*. *tobetsuensis* were designed using the sequence of the *dnaK* gene in the region displaying a high degree of polymorphism (positions 424–1048) [48]. In addition, the distribution and frequency of six species of oral *Veillonella* in the periodontal pockets were recently clarified by PCR using these primer sets and those of our previous study [21]. Thus, our previous study demonstrated that these species-specific primer sets were effective in identifying oral *Veillonella* at the species level, more so than sequence analysis of housekeeping genes. However, these previous procedures used three steps (a two-step PCR with species-specific primer sets for five *Veillonella* species and PCR with species-specific primer pair for *V*. *tobetsuensis*) to define all species of oral *Veillonella*. This study describes the establishment of the first successful one-step PCR method, using species-specific primer sets for the highly variable region of the *rpoB* gene of six species of oral *Veillonella* (positions 2,500–3,100). This one-step PCR method with species-specific primer sets is easier and more effective than previous PCR methods with species-specific primer sets and pairs for oral *Veillonella* described above.

For faster and more convenient identification of the six *Veillonella* species, our pilot study used a multiplex PCR method and was tested in many conditions, simultaneously using all species-specific primer sets and DNA templates of six *Veillonella* species in a single PCR mixture. Only the PCR product of VF and DISR, specific for *V*. *dispar*, could not be confirmed (Data not shown). Although the reason was not clarified, it was suspected to be due to the sensitivity of DISR. The specific primer set for *V*. *dispar*, VF and DISR, showed lower sensitivity than the other sets (Fig 3). Therefore, a one-step PCR method, with species-specific primer sets for six species of oral *Veillonella*, was developed in this study.

According to the results of Tables 2–4, *Veillonella* species had a tendency to be detected in subjects who had poor oral hygiene compared to those with good or moderate oral hygiene. However, the CFU count of all bacteria in tongue biofilms had no relationship with oral hygiene status. It was suggested that the appearance of *Veillonella* species in tongue biofilm of Thai children would be one of the indexes for deteriorating oral hygiene.

In our previous study, the distribution and frequency of oral *Veillonella* at a species level in the tongue biofilm of Japanese healthy young adults (Twenty-seven subjects: 12 males and 15 females, age: 20s) were investigated [12]. In that study, all subjects had *Veillonella* species (more than 0.6 x 10^6^ CFU/mL), and the number of isolates was 416 from all subjects. Tongue biofilm samples from these subjects could be divided into two groups based on the distribution and frequency of five species of oral *Veillonella*. In one group, *V*. *rogosae* was predominant, while the other group consisted of mainly *V*. *dispar* and *V*. *atypica*. Beighton et al. [11] also reported the predominant cultivable *Veillonella* species in tongue biofilm of healthy adults (eleven subjects: the ratio of gender and range of age were not shown) in the UK. They also demonstrated that *Veillonella* species were isolated from all subjects, and the total number of *Veillonella* isolates was 253. In addition, they reported that the predominant species in tongue biofilms were *V*. *atypica*, *V*. *dispar*, and *V*. *rogosae*. In this study, only 10 of 89 subjects had *Veillonella* species, and the total number of isolates was 101 from 10 subjects. The ratio of *Veillonella* isolates was clearly low compared to these previous reports. As described in the introduction, it was reported that *Veillonella* species have a central role in biofilm formation as the early colonizer [32]. The results herein may be due to country- or age-specific differences, in which case, other oral bacterial species may have central roles as the early colonizer to form the biofilm and to cause various oral infectious diseases. As the source of these differences was not clarified here (for example, whether it is due to country- or age-specific differences), further studies are needed to investigate the distribution and frequency of *Veillonella* species from tongue biofilms of children in other countries including Japan.

*V*. *rogosae* was also isolated as the predominant species in tongue biofilms in this study. In addition, *V*. *parvula* and *V*. *denticariosi* were not isolated at all. Similarly, Beighton et al. [11] reported that *V*. *parvula* was isolated from only one subject while *V*. *denticariosi* was not isolated from any subjects in their study. In our previous study [12], *V*. *parvula* was isolated from four subjects and *V*. *denticariosi* was isolated from only one subject. Taken together, it appears that the distribution and frequency of *Veillonella* species in tongue biofilm have generational and universal commonality. In this study, *V*. *tobetsuensis* was isolated from only one subject. In addition, in our previous studies [12, 48], 12 strains of *V*. *tobetsuensis* were isolated from tongue biofilms of 5 of 27 subjects. Here, it was also considered that *V*. *tobetsuensis* is a minor species in the tongue biofilm. There are likely to be several factors that influence these differences in distribution and frequency of *Veillonella* species in tongue biofilms.

PCR products could not be identified in 40 strains, using species-specific primer sets for oral *Veillonella* species, but these 40 strains produced PCR products with the *Veillonella* genus-specific primer set used in the present study. This confirms that these 40 strains were members of the genus *Veillonella*. Although these 40 strains could not be classified as any of the established oral *Veillonella*, these results suggest the possibility that other *Veillonella* species also inhabit the human oral cavity.

In addition, according to the results of clonal analysis, although the representative 11 strains in the 40 unknown strains showed similar sequences with *V*. *dispar*, they formed distinct taxa with robust bootstrap support (100 and 95.9%) in the *rpoB* tree (Fig 4). These 40 isolates may be members of novel oral *Veillonella* species. Furthermore, there are at least two novel *Veillonella* species in the 40 unknown strains based on the evolutionary tree of *rpoB* gene (Fig 4). However, the sequence analysis of other housekeeping genes, such as 16S rDNA, *dnaK* and *gyrB* genes, are required to propose the novel species of genus *Veillonella* [5–7, 44–46]. At present time of writing this manuscript, the sequence analysis of other housekeeping genes of these representative 11 unknown strains are in the process.

In this study, a novel one-step PCR method with species-specific primer sets for oral *Veillonella* was established using a partial sequence of the *rpoB* gene. Moreover, this is the first report of the use of species-specific primer sets for determining the distribution and frequency of oral *Veillonella* in tongue biofilm of Thai children. However, as described above, further studies are needed to discuss the country- or age-specific differences in the distribution and frequency of oral *Veillonella*. The study of the distribution and frequency of oral *Veillonella* by using the tongue biofilms in other countries including Japan will be investigated in near future.
